# Novel, static, permanent spacers to treat chronic knee periprosthetic joint infections

**DOI:** 10.1007/s00264-023-05884-w

**Published:** 2023-07-08

**Authors:** Shuailei Li, Yanyan Meng, Jiankang Pan, Yongqiang Sun

**Affiliations:** 1 Henan Luoyang Orthopedic Hospital (Henan Provincial Orthopedic Hospital), Zhengzhou, 450000 Henan China; 2 Department of Neurology, Zhengzhou Central Hospital, Zhengzhou, 450000 Henan China

**Keywords:** Spacers, TKA, Knee arthrodesis, Periprosthetic joint infections

## Abstract

**Purpose:**

To discuss a static and permanent spacer in the treatment of chronic periprosthetic knee infection. Methods

In this study, patients who were diagonised with chronic periprosthetic knee infection and not appropriate to undergo revision operations were included and were treated with static and permanent spacers. Infection recurrence rate was recorded, Visual Analogue Scale (VAS) score and Knee Society Score (KSS) were used to record patients’ pain and knee function before the operation and at the final follow-up (minimum 24 months).

**Results:**

Fifteen patients were identified for this study. Pain and function were significantly improved at the latest follow-up evaluation. One patient had a recurrent infection and underwent amputation. No patients had signs of residual instability at the final follow-up evaluation, and no breakage or subsidence of the antibiotic spacer were identified at the final radiographic follow-up evaluation.

**Conclusion:**

Our study provided evidence that the static and permanent spacer was a reliable salvage procedure to treat periprosthetic knee infection in compromised patients.

## Introduction

Periprosthetic joint infection (PJI), occurring in 1–3% of patients, is one of the most catastrophic complications following total knee arthroplasty (TKA) [1]. Though there were many management options, the outcome still remains unsatisfactory with reinfection rate was reported to be up to 23% [2]. Debates have long existed on effective way of managing failure of septic revision surgery. Knee arthrodesis, commonly achieved by external fixation, compression plating, or intramedullary nailing, etc., reserved as a limb salvage strategy for PJI, has shown to be a viable option [3, 4]. Previous studies [5–8] have reported a non-union rate of 7–70%, 0–50%, and 0–26% in patients underwent knee arthrodesis by external fixation, compression plating, and intramedullary nailing, respectively. The mean limb-length discrepancy in patients underwent knee arthrodesis by external fixation or intramedullary nailing was revealed to be approximately 3.2–4.5cm.

Temporary antibiotic-loaded cement spacer, traditionally used as an interim method in two-stage revision, has advantages in both infection eradication and joint mobilization retention. Recently, it was reported that it could also be retained and functioned as a permanent solution for PJI with adequate satisfactory rate [9, 10]. Choi et al. [10] described series of patients who did not receive reimplantation by unplanned retention of spacer and were followed up more than 43.8 months, all show no evidence of recurrent infection, and one of seven failed due to spacer loosening. We hypothesized if a static spacer could be used in complex cases as modular and permanent intramedullary nailing, once it was strengthened. In this study, we presented a new technique using static spacers for knee arthrodesis that not only delivered high-dose local antibiotic, but also provided stability of the knee. Our goal was to determine if such technique would eradicate infection and improve postoperative pain and knee function as effective as it was reported in the literature.

## Materials and methods

We retrospectively reviewed one hundred and three patients who underwent treatment for infected TKA [11] from September 2019 to July 2020 at our institution. The study was approved by the Ethics Committee of our hospital. Fifteen patients (9 females [60%] and 6 males [40%]) who underwent static spacer implantation were identified and included in this study. All patients were followed with minimum of 24 months (range, 24–34 months). No patients were lost to follow-up. The mean age of the patients at the time of index surgery was 79.5±14.6 years (range, 75–83 years). All the patients had one or more co-morbidities. Ten patients (66.7%) had arterial hypertension, nine patients (60%) had diabetes, eight patients (53.3%) had rheumatoid arthritis, seven patients (46.7%) had coronary artery disease, seven patients (46.7%) had cardiac insufficiency, and five patients (33.3%) had kidney diseases. Twelve patients (80%) had sinus tract. The diagnosis of PJI was based on the Musculoskeletal Infection Society and the Infectious Diseases Society criteria [11, 12]. The inclusion criteria were patients with one or more of the following conditions: refusing repeated debridement, severe bone loss, extensor mechanism deficiency, too fragile to undergo multiple surgeries, at high risk of recurrent infection.

According to McPherson’s classification [13], all patients identified for this study had infection type III, all the patients were systemic host grade C, eleven patients (73.3%) had local wound grade 2, and four patients (26.7%) had local wound grade 3. The most common indication for permanent static spacers was compromised patients with severe bone loss refusing repeated debridements. Each patient had an average of 2.8 prior procedures (range, 2–6). Joint fluid and tissue cultures were negative in four patients (26.7%). The most prevalent organism isolated from infected revision surgeries were coagulase-negative *Staphylococcus* (5 patients [33.3%]), followed by methicillin-resistant *S. aureus* (3 patients [20%]), and methicillin-sensitive *S. aureus* (3 patients [20%]). Two patients had extensor mechanism deficiency. VAS pain score and Knee Society Score were recorded at preoperation and the most recent follow-up. Infection control was according to Delphi-based international multidisciplinary consensus criteria [14]: (a) no clinical signs of infection, characterized by a healed wound without sinus tract, drainage, or significant pain; (b) no need for additional surgeries intervention for infection after spacer placement; (c) no occurrence of PJI-related mortality; (d) suppressive treatment of less than 6 months. Stability was defined as a clinically stable knee and no radiological sings of breakage or subsidence of antibiotic spacers.

### Surgical technique

The previous incision was used whenever possible. Infected implants were all removed. Infected tissues were collected for cultures and microbiological examination, cement, and bones were meticulously debrided, followed by a complete synovectomy. The canals were irrigated to remove necrotic material and infected tissues.

**Fig. 1:**
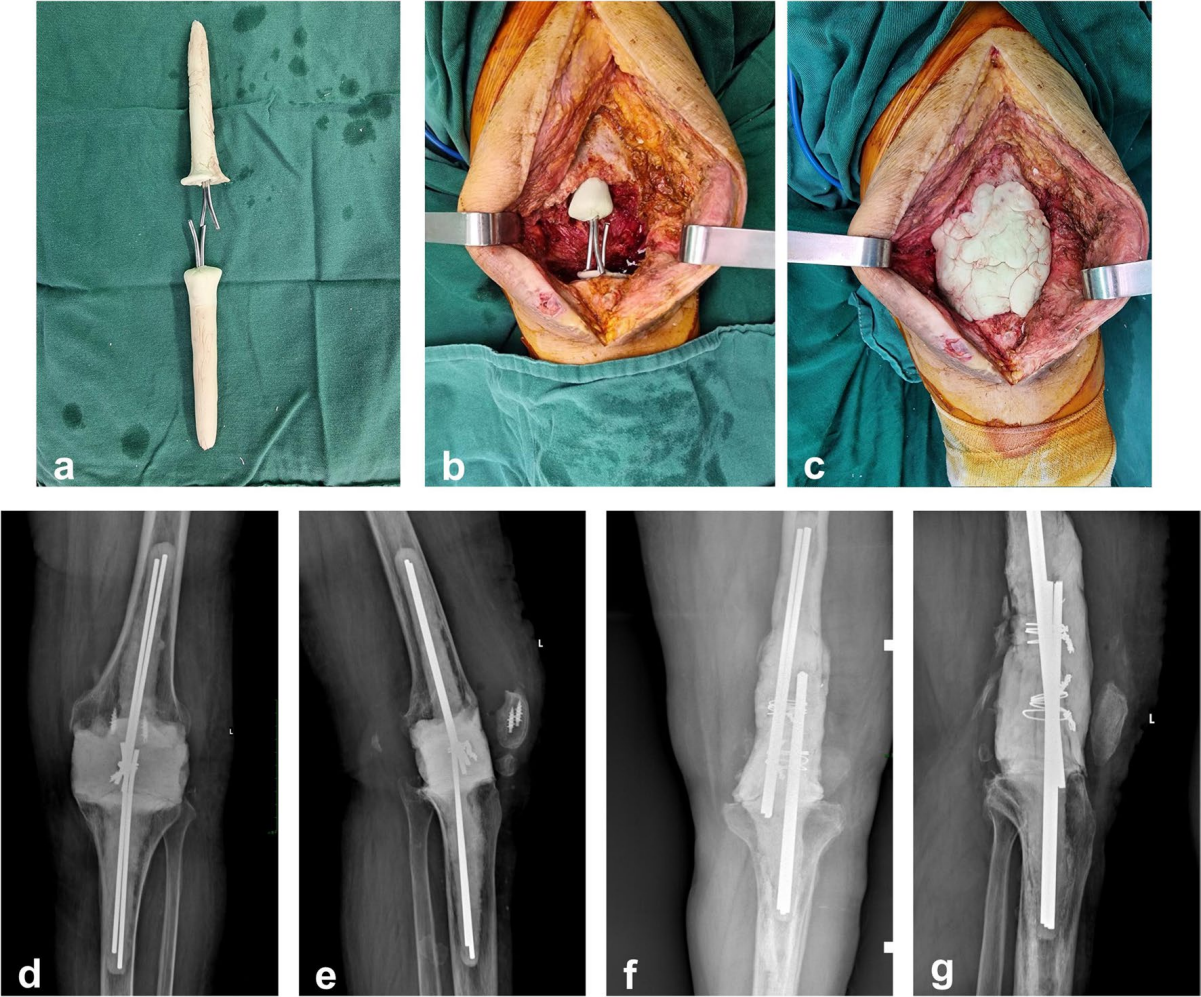
**a**. Self-constructed intramedullary cemented rods. **b** intramedullary cement rods inserted tightly into the femur and tibia. **c** The gap was filled with cement. **d**, **e** A 79-year-old woman was diagnosed as chronic knee periprosthetic joint infections, her past medical history is positive for diabetes and rheumatoid arthritis. She had undergone twice debridement, presented with sinus tract and knee extensor failure. Static and permanent cement spacers was implanted. Anteroposterior and Lateral view of Radiograph of the spacer at 2.4-year follow-up. **f**, **g** An 80-year-old male was diagnosed as chronic knee periprosthetic joint infections, his past medical history is positive for rheumatoid arthritis and atrial fibrillation. There was sever bone defect during the second debridement. Static and permanent cement spacers was implanted. Anteroposterior and Lateral view of radiograph of the spacer at 2-year follow-up

Polymethyl methacrylate (PMMA) was hand-mixed with antibiotic powder (typically 2 g of vancomycin and 2.8 g of tobramycin/per 40 g of PMMA). A self-made intramedullary cemented rod was reinforced by two or three 3.5-mm Steinman pins. In order to tight together the two cement robs in the following steps, the pins were not completely covered by cement. The size and length of cemented rod was made based on the size of the metaphysis cavities of the femur and tibia. Once the size of intramedullary cement rod was confirmed and the cement was fully polymerized, the cement rod was encased by additional bone cement in the doughy phase, then they separately inserted tightly into the metaphysis of the femur and tibia. The knee was extended and the portions of the Steinman pins that were uncovered by cement were overlapped. The overlapped Steinman pins were tightened together by wire, and the gap between the femur and tibia was filled with additional cement. The knee joint was held at 5° flexion and 5° valgus under maximum longitudinal tension until the bone cement was fully polymerized (Fig. 1). All patients were encouraged to mobilize early after surgery, no braces were required. Patients were given intravenous antibiotics postoperatively for two weeks, then changed to dual oral antibiotics for four weeks according to culture results and sensitivities.

### Statistical analysis

Statistical analysis was performed using GraphPad Prism 8. The Wilcoxon signed-rank test was utilized to compare the pre-operative knee joint status with the status at the final follow-up evaluation. Differences with a *p*-value <0.05 were considered statistically significant.

## Results

Pain at rest and knee function were significantly improved at the final follow-up evaluation (Table 1). The visual analogue scale (VAS) score improved from a preoperative median value of 6.4±1.7 points to a median value of 1.7±0.9 points at the final follow-up evaluation (*p*=0.032). The average Knee Society functional score improved from a preoperative value of 14.5±4.8 points to a value of 32.5±10.5 points at the final follow-up evaluation (*p*< 0.001). The average Knee Society clinical score improved from a preoperative value of 39.2±8.4 points to a value of 73.3±10.8 points at the final follow-up evaluation (*p*< 0.001).

**Table 1:** Pre- and post-operative pain and function

	Pre-operation	Last post-operation	*P*
VAS pain score	6.4±1.7	1.7±0.9	0.032
Knee Society functional score	14.5±4.8	32.5±10.5	<0.001
Knee Society clinical score	39.2±8.4	73.3±10.8	<0.001

Six patients died from unrelated causes within one year postoperatively. One of 15 patients (6.7%) had a recurrent infection 1.5 years after the index operation and was treated with an above-the-knee amputation. Other complication was delayed wound healing in two patients (13.4%).

No patients had signs of residual instability at the final follow-up evaluation. There was no breakage or subsidence of antibiotic spacers, and no signs of progressive bone loss at the final radiographic follow-up evaluation.

## Discussion

For patients who had relatively low demand in physical activity and/or with severe bone loss, especially those too frail to undergo repeated operations and refusing repeated debridement, cement spacer may be a long-lasting construct for knee arthrodesis in compromised patients. Choi et al. [10] revealed a successful rate of 86% in a cohort of patients who did not receive reimplantation by unplanned retention of spacer at mean follow up over 43.8 months, with one out of seven patients failed due to spacer loosening. Jan Lesenský et al. [15] reported 11 patients who had bone tumours of femur and underwent permanent cement spacer intercalary reconstruction, while only two patients experienced mechanical failure at mean follow-up of 52 months.

Although static spacer was the preferred option in a host with poor soft tissues and extensive bone loss [16], it was associated with relatively high risk of mechanical complications. Faschingbauer et al. [17] reported in his study on patients developed chronic PJI after primary TKA that 10.5% of these patients with static spacer placement developed mechanical complication, most frequently a fracture of the tibia or femur. However, it was demonstrated more stable spacer could be obtained if it could be strengthened. Sara Scarponi et al. [18] reported no incidence of mechanical complication at minimum follow-up of two years in their study of PJI patients treated with static spacer which was strengthened by cementless modular intramedullary nail to achieve knee arthrodesis. Kotwal et al. [16] reported in a cohort of 58 medically compromised patients who were treated by similar approach, followed up for an average time of 29.4 months, no patient underwent revision for mechanical complications.

The present study revealed no signs of residual instability in any patient and a low recurrent infection rate of 6.6% (1/15) at the final follow-up. The recurrent infection rate in our study was lower than the reported results in other studies using cement with intramedullary nailing [16, 18, 19], in which recurrent infection rate ranged from 10.5 to 23%. Potential reasons could be as follows: first, antibiotic-impregnated bone cement filled serious bone defect and release more antibiotic to eradicate infections. Second, two handmade intramedullary cement rods, not only provided additional stability but also more greater concentration antibiotic to eradicate infections. These results indicate this static and permanent spacer could provide satisfactory clinical outcomes and may be an optimal alternative for patients with recurrent infections who are reluctant to undergo repeated debridement operations.

There were several limitations in our study, as follows: first, the sample size was small. PJI is a rare event, and 103 patients underwent treatment for infected TKA in our institution from September 2019 to July 2020, and only 15 patients were treated with arthrodesis; second, absence of a control group and the outcomes of patients was compared to the contemporary literature.

## Conclusion

This study shows that this static and permanent spacer with cement intramedullary nails is a safe and effective treatment both in controlling PJI and providing satisfactory functional outcomes, which may offer a reliable salvage option to treat PJI of the knee in selected cases.

## Data Availability

The authors confirm that the data supporting the findings of this study are available within the article
